# Multi-bandgap Solar Energy Conversion via Combination of Microalgal Photosynthesis and Spectrally Selective Photovoltaic Cell

**DOI:** 10.1038/s41598-019-55358-6

**Published:** 2019-12-12

**Authors:** Changsoon Cho, Kibok Nam, Ga-Yeong Kim, Yeong Hwan Seo, Tae Gyu Hwang, Ji-Won Seo, Jae Pil Kim, Jong-In Han, Jung-Yong Lee

**Affiliations:** 1 School of Electrical Engineering, Korea Advanced Institute of Science and Technology (KAIST), Daejeon, 34141 Republic of Korea; 2 Department of Chemical and Biomolecular Engineering, Korea Advanced Institute of Science and Technology (KAIST), Daejeon, 34141 Republic of Korea; 3 Department of Civil and Environmental Engineering, Korea Advanced Institute of Science and Technology (KAIST), Daejeon, 34141 Republic of Korea; 4 Department of Materials Science and Engineering, Seoul National University, Seoul, 08826 Republic of Korea; 5 Present Address: Dresden Integrated Center for Applied Physics and Photonic Materials (IAPP), Technische Universität Dresden, Nöthnitzer Straße 61, Dresden, 01187 Germany; 6 Present Address: Agency for Defense Development, Daejeon, 34188 Republic of Korea

**Keywords:** Microbiology, Environmental sciences, Energy science and technology

## Abstract

Microalgal photosynthesis is a promising solar energy conversion process to produce high concentration biomass, which can be utilized in the various fields including bioenergy, food resources, and medicine. In this research, we study the optical design rule for microalgal cultivation systems, to efficiently utilize the solar energy and improve the photosynthesis efficiency. First, an organic luminescent dye of 3,6-Bis(4′-(diphenylamino)-1,1′-biphenyl-4-yl)-2,5-dihexyl-2,5-dihydropyrrolo3,4-c pyrrole -1,4-dione (D1) was coated on a photobioreactor (PBR) for microalgal cultivation. Unlike previous reports, there was no enhancement in the biomass productivities under artificial solar illuminations of 0.2 and 0.6 sun. We analyze the limitations and future design principles of the PBRs using photoluminescence under strong illumination. Second, as a multiple-bandgaps-scheme to maximize the conversion efficiency of solar energy, we propose a dual-energy generator that combines microalgal cultivation with spectrally selective photovoltaic cells (PVs). In the proposed system, the blue and green photons, of which high energy is not efficiently utilized in photosynthesis, are absorbed by a large-bandgap PV, generating electricity with a high open-circuit voltage (*V*_oc_) in reward for narrowing the absorption spectrum. Then, the unabsorbed red photons are guided into PBR and utilized for photosynthesis with high efficiency. Under an illumination of 7.2 kWh m^−2^ d^−1^, we experimentally verified that our dual-energy generator with C_60_-based PV can simultaneously produce 20.3 g m^−2^ d^−1^ of biomass and 220 Wh m^−2^ d^−1^ of electricity by utilizing multiple bandgaps in a single system.

## Introduction

In addition to the electricity generated from solar and wind power, biofuel is an attractive renewable energy source, especially for systems such as transportation, which require liquid forms of energy. The aquatic microalgal biomass is considered to be one of the best-suited feedstocks for this purpose, and it does not require arable land area^1–3^. Moreover, a wide range of the potential applications of the microalgal biomass (food, medicine, agriculture, etc.) makes it more promising in the view of marketability. However, to become economically viable for mass production, the fundamental issue of limited biomass yield, although one-order higher than terrestrial plants, must be resolved.

**Figure 1:**
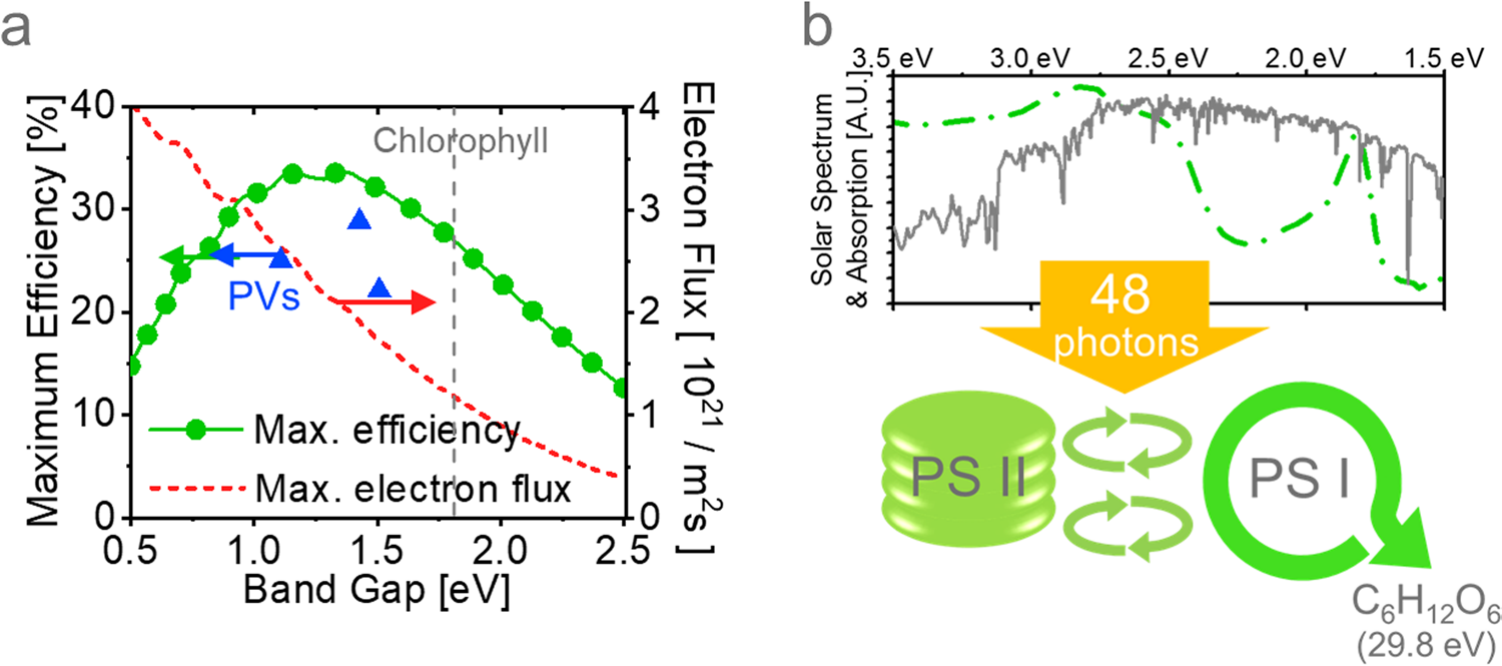
Theoretical efficiency. (**a**) Theoretical maximum power conversion efficiency (temperature: 300 K) and electron flux of semiconductors with various bandgaps under AM 1.5 G illumination. (**b**) Spectra of AM 1.5 G and chlorophyll absorption (top) and simplified photosynthesis model (bottom).

As a process to convert sunlight into a utilizable form, photosynthesis has a similarity to the photovoltaic devices (PVs); however, photosynthesis for terrestrial or microalgal biomass production suffers from the limited power conversion efficiency (PCE), approximately one order lower than that of PVs^4^, restricting the productivity per area. In chlorophyll, which is to be considered a semiconductor with a band-gap (*E*_g_) of 1.78 eV, infrared (IR) photons below the band-gap are not absorbed (53%) and the corresponding maximum electron flux is 1.2 × 10^21^ m^−2^ s^−1^ under 1 sun, as shown in Fig. 1a. In this case, the maximum power conversion efficiency (PCE) reaches only 27%, and the excess energy of the absorbed visible photons is lost as heat. This PCE is lower than the maximum achievable value of 34% at the optimal band-gap of 1.34 eV, called the Shockley-Queisser limit^5,6^. Moreover, in the photosynthesis process depicted in Fig. 1b, through photosystems (PSs) I and II, 48 photons are consumed to produce one molecule of glucose (C_6_H_12_O_6_) with a chemical energy 29.8 eV. The corresponding maximum photosynthesis efficiency (PE) becomes [(1.2 × 10^21^ m^−2^ s^−1^) × (29.8 eV/48)/(1000 W m^−2^)] ~ 12%. In reality, as not all the absorbed photons enter the PSs, it is known that at least 57 photons^7–9^ are consumed per glucose molecule, and the maximum PE becomes ~10% (assuming no loss during the conversion from glucose into real biomass consisting of diverse molecules). While diverse single-junction PVs, such as GaAs (*E*_g_ ~ 1.4 eV), crystalline silicone (c-Si, *E*_g_ ~ 1.1 eV), and perovskite (*E*_g_ ~ 1.5 eV) -based PVs, have achieved high PCEs of more than 70% of their theoretical limits, as shown in Fig. 1a^10^, the PEs of outdoor microalgae cultivation under sunlight are reported to be only ~4% for photobioreactor (PBR) systems^11–15^ and 3- to 5-fold lower for open pond systems^3^ in warm locations, far below their theoretical limit, implying significant opportunity for further technical improvement. It should be noted that some of the previous reports^13,14^ chose a different definition of PE and did not count IR photons as inputs.

To improve the low PE and produce more bioenergy, controlling the *quality* of light is an attractive strategy. Although blue photons have higher energy than red photons, the absorption of blue light is inefficient during photosynthesis because (i) the excessive energy of the blue photons (2.5–3.1 eV) is lost as heat in PS I or II with fixed bandgaps (~1.8 eV), (ii) the quantum efficiency of microalgal photosynthesis is known to be lower for blue photons than for green and red photons^16–20^, and (iii) the absorption of those high-energy photons may cause photoinhibition^21–23^. Moreover, there have been a few studies^24–28^ reporting that microalgae cells cultivated under red light contain more lipids, which are used for fuel production, than those under blue light, while further investigation would be required to reveal the mechanism and quantify the relationship. Accordingly, direct exposure of microalgae to the full spectrum appears to be suboptimal for utilizing solar energy and the available land area.

For those reasons, there have been attempts to down-convert blue or green photons to red photons by adopting luminescent materials^11,27–34^. For example, L. Wondraczek *et al*. reported a >20% enhanced photosynthesis rate by adopting a photoluminescent phosphor^27^. In a similar manner, H. Amrei *et al*. presented further enhancement of microalgal biomass productivity up to 74% using organic luminescent dye^33^. The improved lipid content of 30–70% using spectral conversion was also demonstrated by Y. Seo *et al*. with 20–40% enhanced biomass productivity^11^. However, despite these achievements that showed the potential and importance of light quality control of microalgal photosynthesis, unsolved issues remain: (i) the spectral information of the light source has been rarely reported and are difficult to compare, (ii) the spectrum and intensity of the light used in these experiments were much different from sunlight and the results may not be reproducible in outdoor conditions, and (iii) quantitative analysis of their effectiveness and general design rules are lacking. In this study, we investigated the optical strategies for solar spectrum engineering of photosynthesis with a controllable AM 1.5G-simulating light source. For the spectral conversion scheme, the optical losses due to the low quantum yield and geometrical light propagation efficiency of luminescent materials were shown to be limiting factors for using spectral conversion materials.

Based on the analysis of the potential and limitations of spectral conversion, we introduce an alternative means of utilizing high-energy blue photons in an add-on device, namely, a dual-energy generator that combines a microalgal photosynthesis system with a high-bandgap photovoltaic (PV) module. This innovative device enables the use of red photons for efficient microalgal photosynthesis and blue photons for high-voltage electricity generation. While there have been a few previous approaches that combined PVs with photosynthesis^35,36^, they consisted of a simple combination of two different systems and there were no synergetic effects from the control of light quality. We aimed to achieve the multiple bandgap effect, possibly overcoming the efficiency limit of single semiconductor system. We use a fullerene-based organic photovoltaic cell with a high bandgap, which is best suited for the spectrum separation of sunlight and the utilization of high-energy photons.

## Results and Discussion

### Spectral conversion: bioreactor adopting luminescent materials

**Figure 2:**
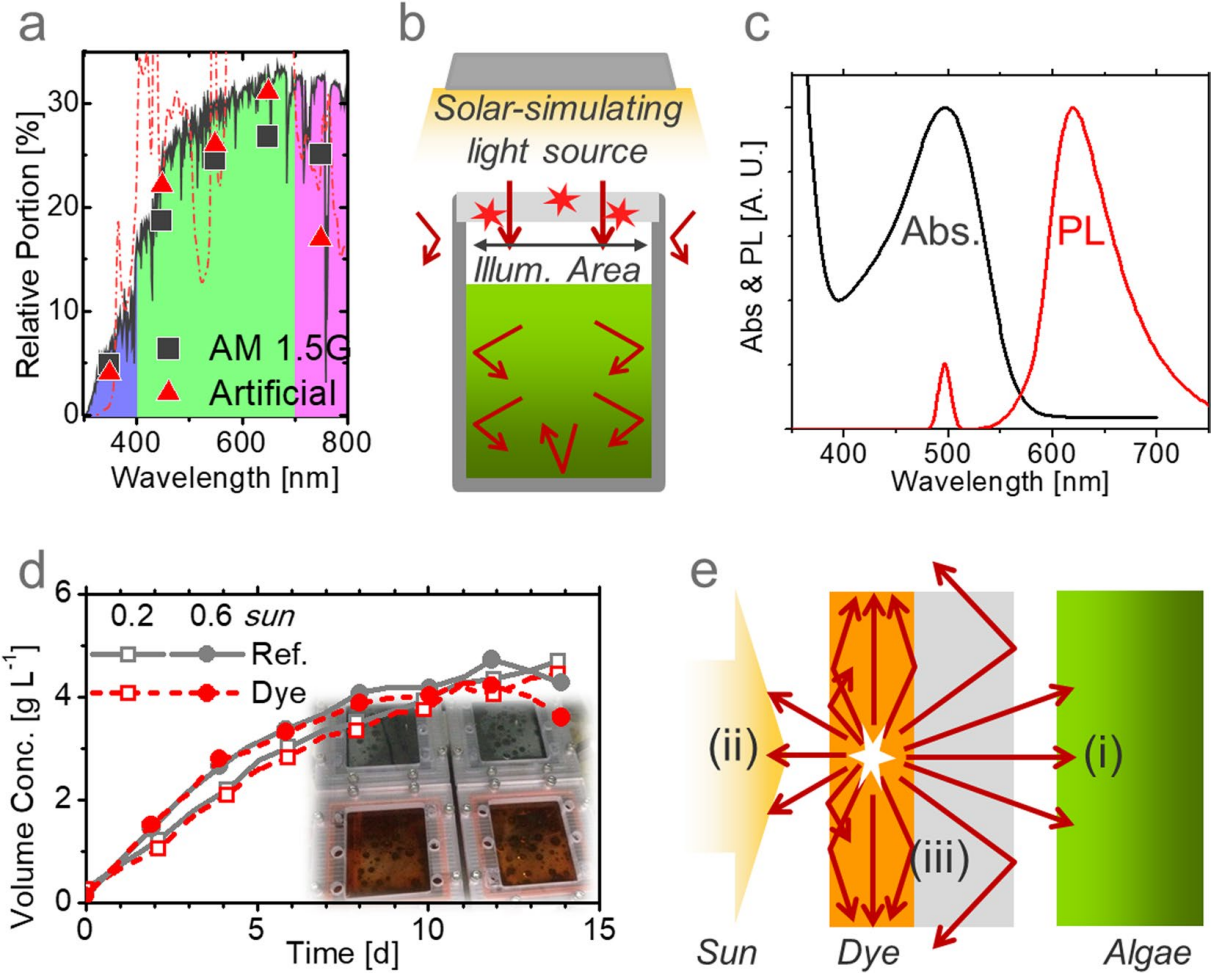
Spectral conversion. (**a**) Number of photons per wavelength (lines in A.U.) and their relative portion (dots) for the real AM 1.5 G (black) and artificial solar-simulating light source (red) used in this study. UV, visible, and IR regions are shown in blue, green, and pink colors, respectively. (**b**) Outdoor-simulating experimental set-up with solar-simulating light source, defined illumination area, periodic boundary condition, and back reflector. (**c**) Measured absorbance and photoluminescence spectrum of D1 in the film state. (**d**) The growth curves of microalgae in volume concentrations with and without D1 coating. (**e**) The schematic illustration of optical loss mechanism for dye-integrated bioreactors.

While most previous reports on the spectral characteristics of microalgal photosynthesis have been based on light emitting diode (LED)-based environments, the quantity and quality of LED were significantly different from the sun and the results may not be applicable to outdoor conditions. For this reason, adopting the appropriate illumination, which mimics the sun in a controllable manner, is crucial to study the optical behavior of an aquatic photosynthesis system in outdoor conditions. Accordingly, we implemented an artificial AM 1.5 G light source, integrating optical filters with a white metal-halide lamp. As shown in Fig. 2a, despite the sharp fluctuations of the measured spectrum of our light source, the integrated proportions of visible photons (400–500 nm, 500–600 nm, and 600–700 nm) roughly matched the AM 1.5 G spectrum, with an error range of 5–15%^9^. Moreover, periodic boundary conditions (PBCs) were applied to simulate large-scale cultivation using smaller bioreactors with limited illumination area. A reflecting metal (aluminum foil or stainless-steel walls) on the sides can prevent energy efflux from inside to outside and energy influx from outside to inside, as depicted in Fig. 2b. In the optical view, the system is identical to the large-scale reactor, where the influx and efflux of light can be compensated. The illumination area was confined by the aperture to strictly define the input energy and prevent overestimation.

Under simulated solar illumination, we examined the change of the algal growth rate by adopting a spectral conversion material. For this experiment, we chose an organic dye, 3,6-Bis(4′-(diphenylamino)-1,1′-biphenyl-4-yl)-2,5-dihexyl-2,5-dihydropyrrolo3,4-c pyrrole -1,4-dione (D1)^37^, as a spectral conversion material, which absorbs photons with wavelengths shorter than 550 nm (i.e. blue and green light) (peak at 495 nm) and emits photons with a photoluminescence (PL) peak at 618 nm (i.e. red light), as shown in Fig. 2c. Such spectral characteristics of optical absorption and luminescence are well-matched with the optical properties of microalgae. While the typical red luminescent dyes show a limited quantum yield of 10–20% in the film state, mainly due to the aggregation and π–π stacking interactions of the molecules in the solid state, D1 was designed to minimize such quenching loss in the film using the phenomenon of aggregation induced emission (AIE)^38,39^. The measured quantum yield of D1 was 24.7% in the film state on a quartz plate^40^, and such outstanding optical properties make D1 an ideal candidate for our proposed system.

Figures 2d and S1 show the results of the microalgae (*Chlorella sp*.) cultivation with and without D1. The light intensity was set to be 0.2 or 0.6 sun with continuous illumination, corresponding to 400 and 1200 μmol m^−2^ s^−1^ of visible photons, or 4.8 and 14.4 kWh m^−2^ d^−1^ of energy. D1 was coated on the polycarbonate cover of the bioreactors with a volume of 500 ml and illumination area of 56 cm^2^. The results of the duplicate reactors were compared with another duplicate reference reactors without dye coatings, as depicted in the inset of Fig. 2d. From their average values, we found no evidence of improved biomass production from the experiments using spectral converting materials. While the average growth rates were already high for the reference reactors (0.41 and 0.49 g L^−1^ d^−1^), with the aid of a high-density photon supply (0.2 and 0.6 sun, respectively), the coating of D1 failed to add productivity and yielded even lower growth rates (0.39 and 0.47 g L^−1^ d^−1^, respectively).

Our results contradict previous research^11,27–34^ that reported enhancements in the biomass productivity by modifying the quality of incident illumination. We speculate that these discrepancies may be due to our different illumination conditions, that is, simulating sunlight. First, organic dyes tend to degrade easily by photooxidation under strong illumination^41^. We observed that the color of D1 after the cultivation was not as dark as the initial state, suggesting degradation. Second, even with identical systems, the light intensity can produce different results. Typical LED light sources have a photon flux far below 0.1 sun and spectra that poorly match chlorophyll absorption spectra. So, the additional illumination with converted red photons may help to enhance the photosynthesis rate. On the other hand, the artificial sunlight we used was as strong as outdoor illumination and contained a sufficient number of red photons to saturate the photosynthesis. Hence, the photon supply may not be a limiting factor for photosynthesis. In previous research^11^, while up to a 40% enhancement of photosynthesis efficiency was achieved with spectral conversion under 0.05 sun, no enhancement was observed in the same system under 0.15–0.2 sun.

In addition to the issues mentioned above, the study of the intrinsic optical limitations of the schemes using luminescent materials would provide further insights into formulating design rules. As depicted in Fig. 2e, the luminescent material absorbs the incident light and re-emits converted photons in dipole form. Subsequently, the emitted photons propagate (i) in a forward direction to the algal solution, (ii) in a backward direction toward the incident light source, and (iii) at the edges guided by total internal reflection (TIR) at the surfaces of the dye and substrate and their interfaces. Assuming an isotropic distribution, the amounts of (i) and (ii) are almost identical and become 1/2*n*^2^/2 per each, where *n* is the refractive index of the emitting material^42,43^. Hence, only 4–11% of the emitted photons for *n* = 1.5–2.5 can be transported to microalgae through (ii), and the photons radiated to (i) escape the system. The other photons are all trapped by TIR, as described in (iii). There is a small chance that the photons will be transported by the re-emission of dye molecules, if the trapped photons are re-absorbed by the dye. In addition to the non-perfect internal quantum yield, such intrinsically limited photon transport efficiency significantly restricts the efficiency of the given architecture. For example, with an internal quantum yield of 25% and light transport efficiency of 11%, a single red photon must be more beneficial than 36 blue and green photons for the photosynthesis process, a seemingly unrealistic requirement, to enhance the overall productivity using this scheme. It should be noted that the reported optical efficiency of red photons is only 10–20% higher than that of blue photons according to their action spectra (*A*_action_(*λ*)).

There exist several possible, but not perfect, strategies to suppress any losses. If the dye-coated substrate directly touches water without an air gap, TIR occurs less and we can obtain a higher optical transmission to the water side (path (i) above), while keeping the undesired backward transmission (path (ii) above) low. If the emitting material is synthesized to have a low refractive index near that of water (*n* = 1.33), as well as a high quantum yield and large Stokes shift, TIR loss can be completely removed, and escape through the backside (~17%) is the only path for losing photons. The use of luminescent materials directly inside the bioreactor in the state of the aqueous solution might be an efficient additional approach to overcome the limitations of film-state dyes. Moreover, control of the dipole orientation and roughening of the film interface can be effective ways to suppress TIR losses. Therefore, while many limitations are present in the current configuration, the development of novel materials and advanced configurations have great potential for improving the biomass productivity of microalgae by modifying the quality of solar illumination.

### Spectral separation: bioreactor combined with spectrally selective photovoltaic cell

Photosynthesis may not be best process for utilizing blue photons. Even with a 100% efficiency scheme to convert blue photons to red photons, the fundamental energy loss of high energy blue photons, of which the excess energy is wasted as heat, is inevitable during photosynthesis because of the fixed bandgap, and limits the PE, as discussed in Fig. 1. Therefore, instead of changing the color of the light, we used an alternative approach to fully utilize the high-energy blue photons.

**Figure 3:**
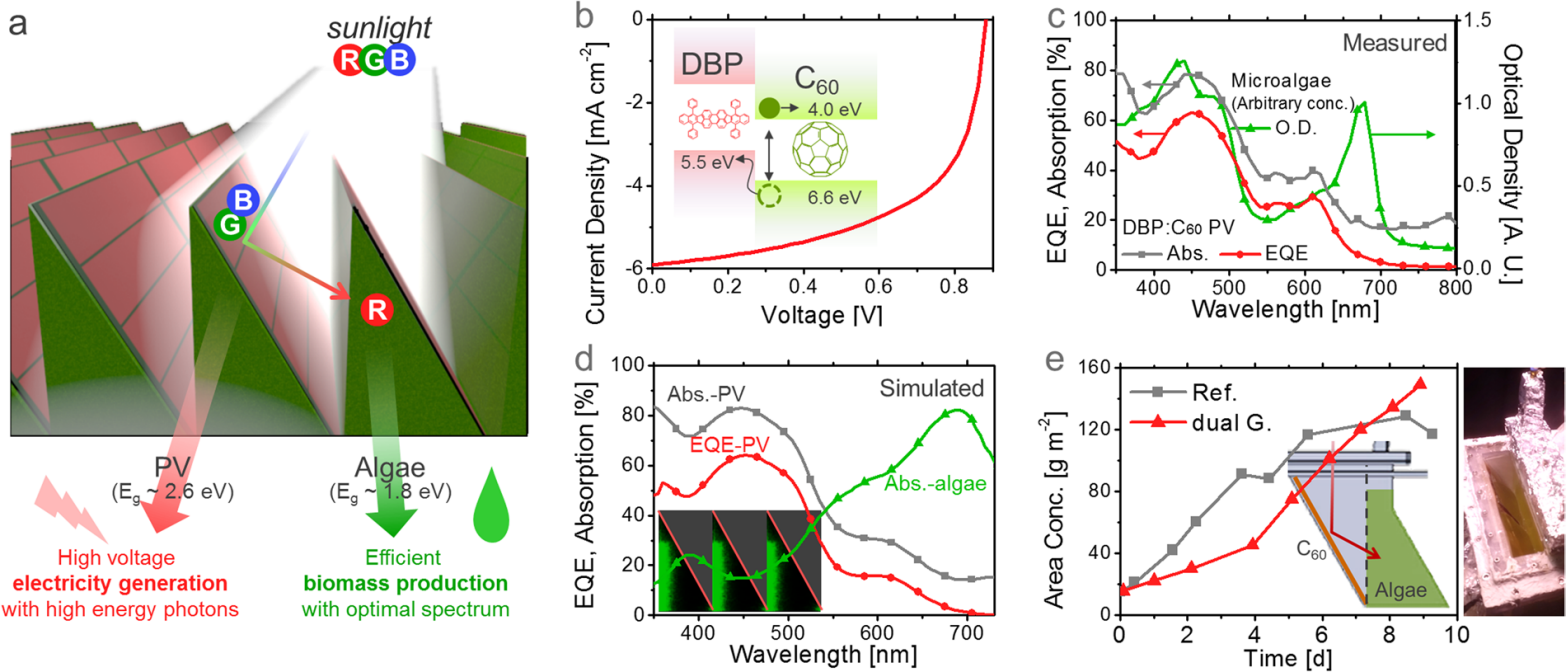
Dual-energy generator. (**a**) Dual-energy generator integrating PVs with microalgal photosynthesis. (**b**) Energy band diagram of the C_60_-based PV and its current density (*J*)-voltage (*V*) curve. (**c**) Separately measured EQE and absorption of a C_60_-based PV and the optical distance of microalgae cells with arbitrary concentrations. (**d**) Simulated EQE (red) and absorption of the C_60_-based PV (grey) and microalgae cells (green) in the dual generator (inset: photosynthetic rate profile inside the configuration with *R*_max_ = 0.30 W g^−1^). (**e**) Measured growth curve of microalgae cells in the growth phase for the flat PBR (gray) and dual-energy generator (red). (Inset: Blueprint and photograph of the dual-energy generator used in our experiment).

Figure 3a illustrates our proposed system, named a *dual-energy generator*. A “spectrally selective PV,” which absorbs only high-energy photons, was installed on one side of a blazed-shape cultivator. In this way, the incident light first impinges on the PVs, and the solar energy can be spectrally split; high-energy (blue) photons are then absorbed by the PV to generate electricity with a high voltage, and low-energy (red) photons are used for biofuel production with the optimal spectral match. The distinctive feature of our approach of combining PVs and photosynthesis is the separation of the spectrum, not the quantity, of incident light with multiple bandgaps. While the theoretical limits of PCE were calculated to be 34% and 27% for semiconductors with bandgaps of 1.34 eV (optimal) and 1.8 eV (chlorophyll), respectively, as shown in Fig. 1a, it is known that the limit increases to ~45% when two different bandgaps exist in a single system and the heat loss of high energy photons is alleviated^44^.

Typically, commercial PV materials, such as c-Si, III-V materials, CdTe, and Cu(In,Ga)Se_2_ (CIGS), have relatively low bandgaps (1.0–1.5 eV), sacrificing output voltage to absorb more red and near-infrared photons. However, in the dual-energy generator, the sacrificing the voltage is not necessary since red photons do not need to be absorbed. As a result, the hybrid system produces both electricity that can be directly consumed and biofuels that can be stored, with minimal interference to the productivity of either process. The bioreactor can also act as a supporting frame and coolant for the PV, and the PV absorbs UV light that is harmful to microalgae, thereby synergistically reducing the energy production cost.

As a spectrally selective PV, we chose a fullerene (C_60_)-based organic PV consisting of tetraphenyldibenzoperiflanthene (DBP) and C_60_, in a ratio of 1:9, whereby the DBP helps to separate the generated charges in C_60_^45^. As shown in Fig. 3b, C60 has a high bandgap of 2.6 eV; thus, it absorbs blue photons with a wavelength shorter than 480 nm. The current density (*J*)-voltage (*V*) characteristics of the PV under 1 sun irradiation exhibited an open-circuit voltage (*V*_oc_) of 0.88 V, which is higher than the *V*_oc_s of conventional c-Si PVs (0.6–0.7 V), at the cost of narrowing the absorption band from 300–1100 to 300–500 nm, and a power conversion efficiency (PCE) of 3.00%, which can generate 220 Wh m^−2^ d^−1^ under 7.2 kWh m^−2^ d^−1^ illumination, corresponding to the typical values for the world’s hottest regions. From an economic perspective, such organic PVs are much cheaper than c-Si PVs^46,47^, and an electricity production of 220 Wh m^−2^ d^−1^ (at 0.07 USD kWh^−1^) is equivalent to a biomass production of 29 g m^−2^ d^−1^ (at 540 USD ton^−1 ^^48^). Covering only ~6% of the total area with this scheme is sufficient to supply electricity for the circulation and dewatering processes of a self-operating cultivation system^48^. If C_60_ is replaced by C_70_ with a lower bandgap (2 eV) in the same PV structure, green photons are absorbed more by the PVs, and the PCE and electric power production further increase to 7.02% and 510 Wh m^−2^ d^−1^, respectively, at the cost of reducing microalgal production, as shown in Fig. S2. Therefore, the types of PV materials chosen must be finely tuned to obtain the desired amounts of electricity and biofuel.

The measured external quantum efficiency (EQE) and absorption of a C_60_-based PV are shown in Fig. 3c with the optical density of microalgae at an arbitrary concentration, measured separately. The absorption spectra of the device were complementary to the absorption spectrum of microalgae. The device is yellow in color because it reflects red and green light. The EQE of ~20% near 600 nm, which is out of the typical C_60_ absorption band, was due to the absorption of DBP.

The performance of the C_60_-based PVs and the bioreactor simultaneously installed inside the dual-energy generator was assessed by optical simulations, as shown in Fig. 3d. While the EQE of the PV was calculated by transfer-matrix formalism (TMF), assuming the internal quantum efficiency (IQE) of 90%^46,47^, the spatial photosynthesis profile of the microalgae (inset of Fig. 3d) was obtained by combining Monte-Carlo simulations with optical response models for photosynthesis^49–51^, fit to our experimental results, as presented in our previous publication^9^. It should be noted that, because photons directly impinge on the PV side first, the calculated PV characteristics of the dual-generator were similar to the measured values of the “PV-only” device, while the calculated algal absorption was highly influenced by the PV properties. According to the simulation results, more than 80% of the blue photons were absorbed by the PVs on the first impingement, and the non-absorbed red photons (near 700 nm) entered the bioreactor and provided energy for photosynthesis. The biomass productivity calculated from the integration of the photosynthesis profile was 15.1 g m^−2^ d^−1^, whereas it was 10.9 g m^−2^ d^−1^ for the C_70_-based PVs (Fig. S2b), where a biomass of 1 g corresponds to 4.9 Wh from its heat value (4.2 kcal g^−1^). Interestingly, the simulated PCE of the C_60_-based PVs in this system was 3.19%, which is, albeit only slightly, even higher than that of the PV-only system. This PCE enhancement can be attributed to the light-trapping effect^9,52–54^ of this system, allowing the PV to recycle the photons that are unabsorbed and reflected by the bioreactor.

We also experimentally implemented the dual-energy generator by combining a bioreactor with C_60_-based PVs, as shown in Figs. 3e and S1. A C_60_ (60 nm)-coated film, which had almost identical optical characteristics to the full PV device, was placed at a titled angle of 30° from the vertical plane. The photons initially pass through the illumination area of 45 cm^2^, and those reflected on the PV enter the bioreactor (*Chlorella vulgaris*) with a cultivation volume of 500 ml. The surfaces other than the illuminated area were covered with aluminum foil to block undesired photon flux. The reference flat bioreactor had a volume of 100 ml and illumination area of 10 cm^2^. Since the reference and our configuration have completely different geometries and illumination areas, the volume productivity is not a suitable figure of merit for comparison. Therefore, we divided the total biomass concentration by the illumination area to obtain the areal biomass productivity. The illumination of 0.6 sun was given with a photoperiod of 12 h:12 h, amounting to a total of 7.2 kWh m^2^ d^−1^.

As shown in Fig. 3e, while the areal biomass productivity for the reference bioreactor was 23.9 g m^−2^ d^−1^ (PE = 1.6%), for the dual-energy generator, it was possible to achieve a biomass productivity of 20.3 g m^−2^ d^−1^ (PE = 1.4%) with an expected electricity production of 220 Wh m^−2^ d^−1^. From the absorption spectrum, it can be calculated that the PV absorbs visible photons of approximately 550 μmol m^−2^ s^−1^ of the total 1200 μmol m^−2^ s^−1^ under 0.6 sun illumination. Thus, the dual-energy generator achieved 85% of the reference biomass productivity with only 55% of the photon numbers, additionally generating high voltage electricity from the remaining high energy photons. Such higher utilization efficiency with a smaller number of photons resulted from the improved quality of red photons and diluted quantity of strong illumination. The summation of 20.3 g m^−2^ d^−1^ and 220 Wh m^−2^ d^−1^ corresponds to the PCE of 4.4% over the entire solar illumination spectrum, which becomes 9.2% if only the visible spectrum is counted as the input.

**Figure 4:**
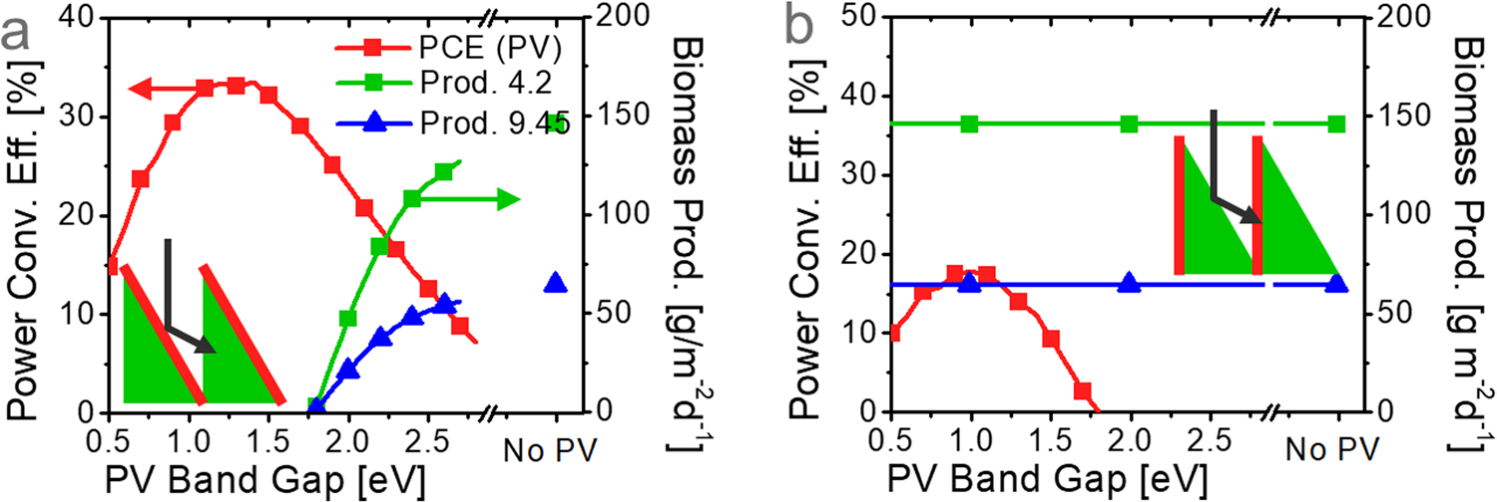
Potential of dual-energy generator. Theoretical maximum PCE (PV) and biomass productivity (containing a bioenergy of 4.2 kcal g^−1^ or 9.45 kcal g^−1^) of the dual-energy generators as a function of the PV band gap under 7.2 kWh m^−2^ d^−1^, when light first impinges on (**a**) PV (red region in the insets) or (**b**) microalgae (green region in the insets). The absorptions of the PV and algae were assumed to be perfect for the photons below their bandgaps.

Figure 4a presents the theoretical maximum energy production of the dual-energy generator as a function of the PV band gap under an illumination of 7.2 kWh m^−2^ d^−1^. As the band gap increases, PV PCE (red) decreases and biomass productivity (green) increases; the biomass was assumed to contain an energy of 4.2 kcal g^−1^ from its measured heat value. For the band gap of 2.6 eV, it is theoretically possible to achieve a PV efficiency of 11.5% and a biomass productivity of 121.2 g m^−2^ d^−1^ simultaneously, assuming 57 absorbed photons generate one glucose (and no further loss occurs) in the photosynthesis process, implying that there remains further room for improvement in our experiment.

Genetic engineering of microalgae to accumulate greater lipid content can result in a higher yield of bioenergy production, allowing a larger amount of biofuel to be extracted from the same amount of biomass. However, since the lipid contains a higher density of energy (9.45 kcal g^−1^) than the other components of the microalgae (e.g. proteins, carbohydrates), higher lipid contents correspond to higher energy densities of the biomass. Thus, a smaller amount of biomass is produced from the same amount of energy produced by photosynthesis. The upper bound of the lipid production can be calculated from the extreme case that all the produced bioenergy is converted into lipid (i.e. an immortal algal culture which produces lipids without any side products). With the heat value of 9.45 kcal g^−1^, the maximum lipid biomass productivity (blue) is only 44% of the green curve with 4.2 kcal g^−1^, as shown in Fig. 4a. This curve indicates the theoretical maximum amount of lipid production under an illumination of 7.2 kWh m^−2^ d^−1^. For example, the maximum lipid productions of 53.9 g m^−2^ d^−1^ and 64.8 g m^−2^ d^−1^ are possible for systems with PVs (*E*_g_ = 2.6 eV) and without PVs, respectively.

Figure 4b shows the theoretical maxima for another type of dual-energy generator, which receives light on the bioreactor side first. If the PV has a bandgap lower than 1.8 eV, it can generate electricity with the photons reflected by the bioreactor side (inset). Contrary to the PV-first system in Fig. 4a, this microalgae-first system can be beneficial for achieving maximum biomass productivity. Since the PV absorbs only IR photons over 700 nm, the maximum PCE is lower and the red curve is shifted to left (i.e. lower value) compared to that in Fig. 4a. The c-Si PV, the most popular PV in the present market, might be a suitable choice for this system due to its bandgap of 1.12 eV near the optimal point.

## Materials and Methods

### Synthesis and characterization of D1

Refer to SI and literature^37,55^.

### Microalgae cultivation with spectral conversion material

*Chlorella sp*. was used for the luminescence experiments. The seed culture was inoculated into a photobioreactor containing 500 ml of BG-11 medium with an illumination area of 56 cm^2^ with initial dry cell weight (DCW) of 0.15–0.25 g L^−1^. It was then cultivated under the following conditions: shaking at 110 rpm, temperature of 32 °C, 0.2 or 0.6 sun of light intensity, and 2% (v/v) of CO_2_ supplementation with 0.4 vvm. The cultivations were prepared in duplicate. The biomass concentration was monitored by measuring the optical density (OD = −log_10_ *Transmission*) and dry cell weight. In particular, the OD was measured at 680 nm with a UV-Vis spectrophotometer (DR 5000, HACH), and the dry cell weight was obtained by Standard Methods^56^.

### PV fabrication and characterization

The current density (*J*)-voltage (*V*) curve was obtained from the full device (area ~0.15 cm^2^) of glass/indium tin oxide (ITO, 75 nm)/MoO_3_ (10 nm)/tetraphenyldibenzoperiflanthene (DBP, OSM, Korea):C_60_ (99.9%, OSM, Korea) (1:9, 50 nm)/C_60_ (10 nm)/bathocuproine (BCP, 99.9%, Lumtec, Taiwan, 7 nm)/Ag (150 nm). The device was fabricated by sequentially depositing MoO_3_, DBP:C_60_, C_60_, BCP, and Ag layers onto a pre-cleaned ITO substrate in a vacuum chamber (<10^−6^ Torr). The *J*-*V* and EQE characteristics of this device were measured using a K201 LAB55 (McScience, Korea) solar simulator and a K3100 IQX (McScience, Korea), respectively. The PCE was obtained at the point of maximum power (*P*_max_ = MAX(−*J* × *V*)) in the *J*-*V* curve, where *P*_max_ was 30 W m^−2^ at *V* = 0.70 V under illumination of 1 sun. The photocurrent density (*J*_ph_) of PV can be obtained by integrating EQE with the equation ∫ *q* EQE(*λ*) Φ_AM1.5G_(*λ*) d*λ*, where *q* is the elementary charge of 1.6 × 10^−19^ C and Φ_AM1.5G_ is the flux of solar illumination (# m^−2^ μm^−1^ s^−1^), equal to AM 1.5 G spectrum over the photon energy (*hc*/*λ*, where *λ* is the wavelength, *h* is the Planck constant of 6.62 × 10^−34^ m^2 ^kg s^−1^, and *c* is the speed of light = 3.00 × 10^8^ m s^−1^). Both the *J*_ph_ and short-circuit current density (*J*_sc_ = *J*_*V*=0_) are shown to be the same (~6 mA cm^−2^) validating the confidence of our measurement.

### Microalgae cultivation with spectrally selective photovoltaic cell

For the dual-energy generator, *Chlorella vulgaris* from the University of Texas (UTEX-265) was cultivated under CO_2_ aeration (5% v/v and 1 vvm) with a fixed volume of 500 ml, pH of ~7, and illumination area of 45 cm^2^ in the water bath (27 °C). The reactors containing BG-11 medium were continuously shaken at 95 rpm. The biomass concentration was determined from the measured OD at a wavelength of 680 nm. The biomass concentration was calibrated by measuring the DCW, which was shown to be 0.52 × OD g L^−1^ (R^2^ = 0.9991 for linear fitting). Deionized (DI) water was added at a rate of 16 ml d^−1^ to compensate for the evaporation of water and maintain the total volume. A heat value of 4.2 kcal g^−1^ of the biomass, which was measured using a calorimeter (Parr-1261, Parr Instrument), was used for calculating the PE.

### Photosynthesis modeling

To model the photosynthesis that occurred in the dual-energy generator, a recently reported custom-made simulation^9^, based on ray-tracing^54,57–60^, was used to obtain the optical absorption profile inside the system. Subsequently, the photosynthesis profile was calculated using the model^49–51^

1 $$PR(x,z)={C}_{volume}\times {R}_{max}\times tanh\,(\frac{\int {A}_{action}(\lambda )\times \#Ph.\,(\lambda ,x,z)\,{\rm{d}}\lambda }{{C}_{volume}\times {R}_{max}}),$$

where *#Ph*.(*λ*, *x*, *z*) is the absorption rate of photons per volume at a given position (*x*, *z*) and wavelength (*λ*), and *A*_action_(*λ*) is an action spectrum indicating the ratio of the photons used for photosynthesis to the total absorption, assumed to be the same as the value for *Chlorella pyrenoidosa*^16,17^ reported previously^9^. A biomass concentration (*C*_volume_) of 1.4 g L^−1^ was used for the calculation based on a typical concentration in the middle of the growth phase in the experiments. The maximum photosynthesis rate per biomass (*R*_max_) was the only fitting parameter, and we selected a value of 0.30 W g^−1^, yielding an overall biomass productivity of ~20 g m^−2^ d^−1^ under 7.2 kWh m^2^ d^−1^, best fit to our experimental values^9^. *#Ph*.(*λ*, *x*, *z*) was obtained by the 2D ray-optical simulation^9,54^ with a measured light extinction coefficient of α = 2.1 cm^−1^. The effects of photoinhibition and change of lipid accumulation as a spectral response were not accounted for this model.

## Conclusions

Understanding the solar spectrum allows the design of more efficient energy conversion systems that use sunlight as an input. We applied spectral engineering to achieve enhanced photosynthesis efficiency of microalgae. Despite their great potentials, the classical approaches that use luminescent materials to convert blue photons to red photons have not yielded enhanced biomass productivity, possibly due to the intrinsic low optical efficiencies. As an advanced scheme, a dual-energy generator that combines spectrally selective PVs and photosynthesis was proposed, utilizing multiple bandgaps of fullerene (2.6 eV) and chlorophyll (1.8 eV) with minimum energy losses. The proposed system experimentally achieved 85% of the reference biomass productivity, while simultaneously producing an expected additional electricity of 220 Wh m^−2^ d^−1^ under the AM 1.5 G illumination of 7.2 kWh m^−2^ d^−1^.

## Supplementary information

Supplementary information

## Data Availability

The datasets generated and/or analysed during the current study are available from the corresponding author upon reasonable request.
