# Supplementary material for: Multi-bandgap Solar Energy Conversion via Combination of Microalgal Photosynthesis and Spectrally Selective Photovoltaic Cell
Source: Sci Rep. 2019 Dec 12;9:18999. doi: 10.1038/s41598-019-55358-6 (PMC6908680; doi:10.1038/s41598-019-55358-6)
Supplement: Supplementary file 1 — Supplementary information [file 41598_2019_55358_MOESM1_ESM.pdf]

Supplementary Information

Multi-bandgap Solar Energy Conversion via Combination of Microalgal Photosynthesis and Spectrally Selective Photovoltaic Cell

Changsoon Cho, Kibok Nam, Ga-Yeong Kim, Yeong Hwan Seo, Tae Gyu Hwang, Ji-Won Seo, Jae Pil Kim, Jong-In Han*, and Jung-Yong Lee*

Synthesis and characterization of D1

3,6-Bis(4′-(diphenylamino)-1,1′-biphenyl-4-yl)-2,5-dihexyl-2,5-dihydropyrrolo3,4-c pyrrole -1,4-dione (D1). [1,2] A mixture of $Pd(PPh_3)_4$ (100 mg, 0.091 mmol), D6C[2] (0.28 g, 0.46 mmol), and 4-[bis(biphenyl-4-yl)amino]phenylboronic acid (BTPA) (0.20 g, 0.69 mmol) in dry tetrahydrofuran (THF) (40 mL) was stirred for 30 min at room temperature under a nitrogen atmosphere. After increasing the temperature of the mixture to 60 °C, an aqueous solution of K_2CO_3 (3.65 M, 5 mL) was added dropwise, and the resulting mixture was maintained at this temperature for 12 h. The reaction mixture was poured into water and extracted with CH_2Cl_2. The combined organic layers were dried over anhydrous $MgSO_4$, after which they were evaporated to dryness. The crude product was purified by column chromatography using CH_2Cl_2:hexane (3:1, v/v) as the eluent to obtain red crystals of T2 (0.35 g, 80% yield). ^1H NMR (500 MHz, CD_2Cl_2), δ (ppm): 7.88–7.89 (d, J = 8.5 Hz 2H),7.76-7.77(d, J = 8.5 Hz, 2H) 7.66-7.70(m, 4H), 7.59–7.62 (m, 6H), 7.55–7.56 (d, J = 8.5 Hz, 4H), 7.40–7.43 (t, J = 7.75 Hz, 4H), 7.29–7.32 (t, 2H), 7.23–7.24 (d, J = 8.5 Hz, 6H), 3.75-3.78(t, J = 7.75 Hz, 2H) 3.69-3.72 (t, J = 7.75 Hz, 2H), 1.52–1.57 (m, 4H), 1.18–1.24 (m, 12H), 0.80-0.82 (m, 6H). ^{13}C NMR (125 MHz, $CDCl_3$), δ (ppm): 163.00, 162.77, 149.06, 147,95, 146.77, 143.60, 140.72, 136.27, 133.89, 132.43, 130.31, 129.48, 128.99, 128.21, 128.13, 127.36, 127.20, 127.06, 126.94, 126.55, 125.76, 125.02, 124.04, 110.40, 109.80, 42.34, 42.14, 31.42, 29.65, 26.62, 26.60, 22.67, 14.17. MALDI-TOF MS: *m/z* 932.3 (100%, M^+). Elemental analysis: Calcd for $C_{60}H_{56}BrN_3O_2$: C, 77.40; H, 6.06; N, 4.51; O, 3.44. Found: C, 77.22; H, 6.17; N, 4.52; O, 3.49.

Bioreactors used in the experiment

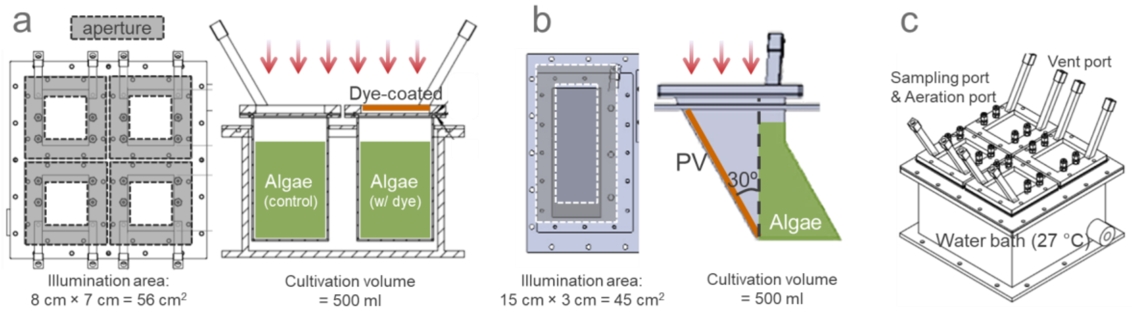

Figure S1. Diagram of bioreactors used in the (a) spectral conversion and (b) dual energy generator experiments. (c) Bioreactors operated in a water bath

Figure S1 shows a diagram of the bioreactors used in our experiments. The temperature was maintained with a water circulation bath, and the light quantity was controlled using apertures with well-defined areas. The water bath was installed on a shaker to keep the cells shaken.

Dual generator using C_{70}-based PVs

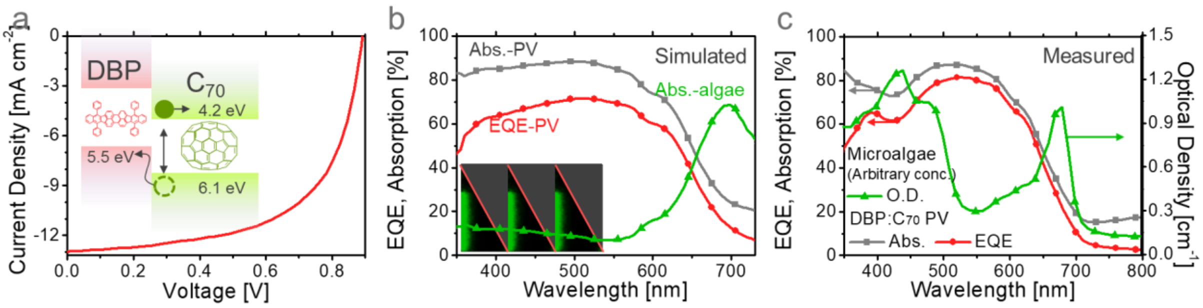

Figure S2. (a) Energy band diagram of a C_{70}-based PV and its current density (*J*)-voltage (*V*) curve. (b) Simulated external quantum efficiency (EQE) (red) and absorption of the C_{70}-based PV (grey) and microalgae cells (green) implemented in a dual generator (inset: photosynthesis rate profile inside the configuration). (c) Separately measured EQE and absorption of the C_{70}-based PV and optical distance of microalgae cells with an arbitrary concentration.

Figure S2 shows the expected performances of the dual-energy generator using a C_{70}-based PV rather than a tetraphenyldibenzoperiflanthene (DBP):C_{60} PV. Compared with C_{60}, C_{70} has a relatively lower bandgap (~2 eV); thus, the PV absorption is shifted to the red spectrum. Due to the trade-off between PV absorption and algae absorption, the measured PV efficiency increased to 7.02% (J_{sc} = 13.0 mA cm^{-2} and V_{oc} = 0.89 V, as shown in Figure S2a) corresponding to 510 Wh m^{-2} d^{-1} under 0.6 sun with a 12 h:12 h photoperiod, and the simulated biomass productivity decreased from 15.1 g m^{-2} d^{-1} to 10.9 g m^{-2} d^{-1} (Figure S2b). The color of the C_{70}-based PV was nearly pure red; therefore, the spectrum of reflected light better matched the absorption spectrum of chlorophyll, although the total amount of photon influx for photosynthesis decreased. Although we used C_{60} in this manuscript, aiming at maximum biomass productivity, we can produce 133% more electricity by replacing C_{60} with C_{70}, sacrificing 28% of the biomass productivity, depending on the purpose of the system.

References

1 Guo, E. Q., Ren, P. H., Zhang, Y. L., Zhang, H. C. & Yang, W. J. Diphenylamine end-capped 1,4-diketo-3,6-diphenylpyrrolo 3,4-c pyrrole (DPP) derivatives with large two-photon absorption cross-sections and strong two-photon excitation red fluorescence. *Chem. Commun.*, 5859-5861, doi:10.1039/b911808j (2009).

2 Zhang, F., Jiang, K.J., Huang, J.H., Yu, C.C., Li, S.G., Chen, M.G., Yang, L.M. & Song, Y.L. A novel compact DPP dye with enhanced light harvesting and charge transfer properties for highly efficient DSCs. *Journal of Materials Chemistry A* **1**, 4858-4863, doi:10.1039/c3ta10618g (2013).
